# Team approach concept in management of oro-facial clefts: a survey of Nigerian practitioners

**DOI:** 10.1186/1746-160X-5-11

**Published:** 2009-05-10

**Authors:** Victor I Akinmoladun, Obitade S Obimakinde

**Affiliations:** 1 Department of Oral and Maxillofacial Surgery, University College Hospital, P.M.B 5013, Ibadan, Nigeria; 2 Department of Oral and Maxillofacial Surgery, University College Hospital, Ibadan, Nigeria

## Abstract

**Background:**

Cleft palate craniofacial teams have evolved across the globe in the last 20 years in compliance with the interdisciplinary concept of management of oro-facial clefts. An interdisciplinary care allows a coordinated treatment protocol for the patient. The objective of this study was to evaluate oro-facial cleft care in Nigeria with particular emphasis on the compliance of the practitioners to the team approach concept.

**Methods:**

A snapshot survey was conducted among specialists that attended the Pan African Congress on Cleft Lip and Palate, at the International Institute of Tropical Agriculture, Nigeria in February 2007.

**Result:**

Sixty three respondents successfully completed and returned the questionnaire for analysis. Mean age of respondents was 43.5 years and the range was 38–62 years.

Male to female ratio was 2.7:1. Oral and Maxillofacial Surgeons and Plastic Surgeons constituted the majority of respondents (38.1% and 22.2%) respectively. Only 47.6% (n = 30) of the specialists belonged to cleft teams. Majority of Oral and Maxillofacial Surgeons and Plastic Surgeons belonged to cleft teams (70% and 63.3% respectively) while speech pathologists and orthodontists were less represented (20% and 36.7% respectively) in teams.

**Conclusion:**

Findings from this study suggests that interdisciplinary care for the cleft patient does not appear to have been fully embraced in Nigeria. This may be a result of several reasons ranging from non availability of the requisite specialists, the relatively young age of cleft care practice in this part of the world to the poor state of infrastructure.

## Background

Craniofacial anomalies, most especially cleft lip and palate are major human birth deformities with worldwide incidence of 1 in 700 and are associated with substantial clinical and psychosocial impact on the society [1]. Little data exist in relation to oro-facial cleft incidence in Nigeria and most African population for several reasons including non availability of reliable birth registers and national statistics.

Management of oro-facial cleft deformities has recently focused on interdisciplinary approach and several descriptions have been referred in the literature [2]. Cleft palate teams have evolved across the globe over the last 20 years in order to provide coordination between different professionals involved in the care of patients with clefts [3]. The specialties involved in orofacial cleft management essentially should include the Orthodontist, Plastic Surgeon, Oral and Maxillofacial Surgeon (OMFS), Otorhinolaryngologist and Speech Pathologist [3-6]. Others such as Audiologist, Paediatric Surgeon and Genetic Counselor or Psychologist have been mentioned in the literature but their services are not universal [3]. An interdisciplinary care allows for the best possible treatment outcome with each member of the team involved in a coordinated treatment protocol for the cleft patient [6].

The American Cleft Palate-Craniofacial Association's Consensus Conference of 1991 postulated that orofacial cleft management is best provided by interdisciplinary team of relevant specialists [7]. It also reported that there is less compliance with team care approach from the developing world. Shortages of professional manpower and socio cultural beliefs of the people are important factors militating against contemporary cleft care in the developing world. It was reported that the African patient exist in a sociocultural matrix which determines the quality of contemporary medical care receivable by such patient [8]. This has a major influence on health behaviour including concept of disease causation, health utilization pattern and relationship with health professionals [1]. Some authors have also reported that volunteer services by surgeons from western world have helped reduce the burden on few specialists available in this part of the world [8,9]. However interdisciplinary cleft care is not usually practiced, this is because volunteer specialists are mainly surgeons who usually carry out single visit surgical repair of oro-facial clefts.

The literature is replete with studies on surgical repair of facial clefts and treatment outcomes but only few studies have stressed the importance of team approach to cleft lip and palate management and the scope of services rendered by each team member. The purpose of this study was to evaluate the practice of the team approach concept and pattern of cleft care practices by the various specialists involved in cleft care practice in Nigeria.

This effort, to the best of our knowledge, is the first attempt at evaluation of team care of the cleft patient in Nigeria.

## Methods

The present study is a questionnaire survey designed to evaluate the compliance of Nigerian practitioners to the team approach concept in management of patients with oro-facial cleft. It was conducted among Nigerian specialists at the Pan African Conference of Cleft Lip and Palate [February 2007]. It was a snapshot survey and the questionnaire was adapted from a previous study by Pannbacker et al [3] [Additional file 1]. Non Nigerian specialists and other participants who are not specialists were excluded from the survey. The questionnaire was designed to evaluate the following: demographic data of respondent, specialty and year of experience, experience in cleft care and involvement in cleft care, scope of services rendered, proportion of patients in different age categories and the types of cleft treated.

Data obtained from the survey was converted to relative values in frequency tables for analysis.

## Results

Of the seventy two questionnaires, sixty three respondents successfully completed and returned the questionnaire for analysis [87.5% response rate]. The age range of the respondents was 38–62 years with a mean age of 43.5 years [median age 42 years, SD 5.425]. The male to female ratio was 2.7:1. Fifty four [85.7%] of the professionals had their postgraduate training in Nigeria while the rest trained in Europe. Of the respondents, 46 [73%] currently practice in Teaching hospitals, 25.4% in Government [non teaching] hospitals while 1.6% works in private hospitals.

Oral and Maxillofacial Surgery [n = 24] and Plastic Surgery [n = 14] had the highest number of respondents [38.1 and 22.2% respectively] followed by Orthodontics with 12.7% [n = 8] [Table 1]. Other specialties were sparingly represented with General Surgery and Restorative Dentistry having the least representation [1.6% each].

**Table 1:** Specialty of respondents

Specialty	Frequency	Percentage
OMFS	24	38.1
Orthodontist	8	12.7
Plastic surgeon	14	22.2
Otorhinolaryngology	5	7.9
Anaesthesia	5	7.9
Speech pathology	2	3.2
Paediatrics	3	4.8
Restorative dentistry	1	1.6
General surgery	1	1.6

Total	63	100

Forty one respondents [65%] were less than10 years post specialization while 27% have more than ten years post specialization experience [Table 2]. The remaining 7.9% [n = 5] did not indicate their post specialization experience.

**Table 2:** Post specialization experience of the respondents in years

Experience (years)	Frequency	Percentage
1–5	16	25.4
6–10	25	39.7
11–15	11	17.5
16–20	2	3.2
> 20	4	6.3
Missing	5	7.9

Total	63	100

**Figure 1:**
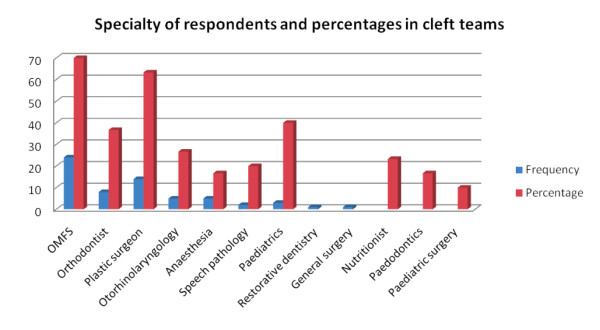
**The figure described the representation of various cleft care professionals on cleft teams.** Only 30 of the 63 participants belonged to cleft team [46.6%].  Of these, 70% had OMFS as team members while 63.3% of such had plastic surgeons as cleft team members. Others are distributed as follows: Orthodontics 36.7%, Otorhinolaryngology 26.7%, Anaesthesia 16.7%, speech pathologist 20%, paediatrics 40%, nutritionist 23.3% , paedodonticss 16.7%, paediatric surgery 10%. These percentages were represented alongside the frequency of each specialty among the participants. Thus OMFS has a frequency of 24, plastic surgery 14, orthodontics 8, otorhinolaryngology 5, anaesthesia 5, speech pathology 2, paediatrics 3, restorative dentistry 1 and general surgery 1. Nutritionists, paedodontics and paedtriatic surgery do not have representation among the respondents, although some respondents claimed that these specialties were members of their respective cleft teams.

Regarding team approach to interdisciplinary cleft care, 30 [47.6%] of the respondents claimed to belong to established cleft teams in their institutions. The result showed that OMFS and Plastic Surgery were the predominant specialties [70 and 63.3% respectively] while Orthodontics was less represented [Figure 1]. Only 36.7% of the respondents who were in cleft teams claimed to have an orthodontist while six [20%] of the respondents had speech pathologists. [Table 3]. Most of the existing teams were relatively young as 80% [n = 24] of those who were in cleft teams indicated that their teams were less than six years in existence.

**Table 3:** Specialties that were represented on cleft teams

Specialty	Frequency	Percentage
OMFS	21	70.0
Orthodontics	11	36.7
Plastic surgery	19	63.3
Otorhinolaryngology	8	26.7
Speech pathology	6	20.0
Nutritionist	7	23.3
Paediatrics	12	40.0
Anaesthesia	5	16.7
Paedodontics	5	16.7
Paediatric surgery	3	10.0

The average patient turnout was eight per month with cleft lip constituting 50–75% of cases seen while patients with isolated cleft palate were in the range of 25–50%. Majority of the patients were children under 3 years of age [50–75%] while those older than six years constitute less than 25% of the patient population. Primary repair of cleft lip and palate were the procedures mostly performed as 90% [n = 40] of the 44 surgeons responded so. Sixteen [25.39%] of the professionals have referred patient with unoperated cleft for prosthetic management. Most of the surgeons [n = 41, 93.2%] indicated that they do not perform pharyngeal flap procedure.

Sixty five percent [n = 41] of the respondents claimed to be involved in researches. Regarding financial support towards cleft care, 27% [n = 17] of the respondents claimed to have some form of financial support presently. The challenges to cleft care reported were finance [75.4%], logistics [34.4%] and sociocultural [41%].

## Discussion

Demography of the respondents revealed a mean age of 43.5 years and most respondents [n = 41, 65.1%] were less than ten years post specialization. This showed that cleft care in Nigerian population is young, though there appears to be an increasing awareness as more core practitioners in oro-facial cleft management are emerging. Furthermore, the dearth of specialists with over ten years experience could be due to the fact that reconstructive surgery [cleft inclusive] did not attract enough trainees in the past [10].

The male: female ratio of 2.7:1 of respondents is comparable to other studies where males are predominant [2,3].

Less than half [47.6%] of the respondents belonged to cleft team, this contrasts sharply with studies from Europe and America where specialists are in cleft-craniofacial teams [2-4,6,11]. Lack of adequate personnel and sociocultural issues are likely problems affecting interdisciplinary cleft care in our environment and furthermore, some health institutions rely on volunteer services from foreign specialists in order to cope with the burden of providing health care services, cleft inclusive [8].

OMFS and Plastic Surgeons were the most frequent specialists in institutions where cleft teams were present, orthodontists and other equally relevant specialists like speech pathologists, otorhinolaryngologists and nutritionists were sparsely represented. The non existence of these specialties in most cleft palate teams makes it difficult to practice the concept of team approach in the management of oro-facial clefts. Rather patients are subjected to primary repair by surgeons while other aspects of care such as speech, orthodontics and other secondary procedures are left unattended to resulting in less optimum outcome.

Furthermore patients with cleft are more likely to present to the OMFS and plastic surgeon since the two specialties are pivotal to the primary surgical repair of oro-facial cleft [12]. The concept of centralized cleft care system has been suggested for the developing world where there is shortage of professional manpower [10]. This entails regional location of facilities, whereby specialists are pooled together. This would more likely result in a complete team composition contrary to decentralization of cleft care, where multidisciplinary management rather than interdisciplinary care is promoted.

Most of the patients seen were less than 3 years of age and 4 [4.4%] of the respondents have treated patients older than 17 years. These findings agree partly with other studies where children constitute majority of the patient population [1,13], however the number of unrepaired adult cases is higher than in the developed world where unrepaired cleft is rare [13]. Lack of health care personnel in some communities, socio cultural beliefs and financial considerations may be responsible for late presentation. Average monthly turnout of eight patients reported by the respondents suggests that the prevalence of cleft lip and palate is perhaps higher in the African population than previously thought.

Primary repair of cleft was the most common procedure done by the surgeons while secondary procedures were reported by 10% [n = 4] of the surgeons. Poor perception on the part of the patients and finance may be two of the reasons responsible for this finding. Moreover most of the respondents were less than ten years post qualification and they may not have adequate exposure and experience regarding secondary procedures.

65% of the respondents engage in research activities. This figure can be explained by the fact that 73% [n = 43] of them practice in teaching hospitals where research is mandatory. If such research activities are focused on various aspects of cleft, indigenous figures on epidemiology and aetiopathogenesis of the condition could soon become available.

In this survey the greatest challenge facing cleft management was finance [75.4% of respondents] as most parents are unable to provide fund for treatment of the cleft child. Funding for cleft management is poor with only 27% [n = 17] of the respondents enjoying some financial support for treatment of their patients. Cleft perhaps does not rank high on the list of the government and Non Government Organisations for attention in a society where malaria and HIV-AIDS constitute major health burdens. Socio cultural beliefs is another challenge to cleft care in this part of the world [5,9].

## Conclusion

In conclusion, findings from this study suggest that interdisciplinary care for the cleft patient is still in its infancy in Nigeria. This may be a result of several reasons ranging from inadequate number of specialists, to the relatively young age of cleft care practice in that part of the world. Although there is sufficient patient population to maintain clinical expertise especially in centers where cleft teams exist, there is still need to encourage training more people to optimize cleft care.

## Competing interests

The authors declare that they have no competing interests.

## Authors' contributions

VIA conceived the study, OSO did the literature search. Both authors jointly prepared the manuscript.

## Supplementary Material

Additional file 1 **Questionnaire on assessment of team approach in management of oro-facial cleft**. The questionnaires were distributed among Nigerian practitioners to assess the level of compliance to management of oro-facial cleft. The data provided by the respondents were analyzed and and presented in the article. Click here for file
