# Supplementary material for: Team approach concept in management of oro-facial clefts: a survey of Nigerian practitioners
Source: Head Face Med. 2009 May 10;5:11. doi: 10.1186/1746-160X-5-11 (PMC2692974; doi:10.1186/1746-160X-5-11)
Supplement: Additional file 1 — Questionnaire on assessment of team approach in management of oro-facial cleft. The questionnaires were distributed among Nigerian practitioners to assess the level of compliance to management of oro-facial cleft. The data provided by the respondents were analyzed and and presented in the article. [file 1746-160X-5-11-S1.doc]

CLEFT LIP AND PALATE QUESTIONNAIRE

Dear sir/ma, this Questionnaire was designed to evaluate some aspects of cleft care amongst specialists involved in management of cleft lip and palate. Kindly respond appropriately to the following questions.

Thank you,

O.S Obimakinde (Department of Oral and Maxillofacial Surgery, UCH Ibadan).

1. Age (years)……, Sex (M/ F)……., Country of practice…………

2. Specialty……………………………

3. Year of qualification as a specialist……………….

4. Where did you undergo specialist training (Tick as appropriate):

- Africa……
- America….
- Europe……

Others (specify)…….

5. Nature of practice:

Government (Non Teaching)…..

Private……

Teaching Hospital…….

6. How long have you been into care of cleft patients?

- < 5 years.
- 5 – 10 years.
- > 10 years.

6. Is there a cleft team in your center? Yes……. No……..

If yes, (i) When was it established? (a) <3 years. (b) 3 – 6 years. (c) >6 years.

(ii) How often does your team meet……..

(iii) List the membership of your team by specialty; …………………….

……………………..

……………………..

……………………..

……………………..

……………………..

……………………...

……………………...

7. On the average how many patients do you attend to each month………

8. Reason for interest in cleft care………………………………………………

9. If you are a surgeon, what is the nature of care you give to the patients?

Primary cleft repair /__/, Secondary repair /__/, Both /__/.

10. Do you currently enjoy any financial support for your patients? Yes….No…..

11. Please circle the approximate percentage of your patient population in each age

Group:

0 - 3 years 0% 1 – 25% 26 – 50% 51-75% >75%

4 – 6 years 0% 1 – 25% 26 – 50% 51 – 75% >75%

7 – 12 years 0% 1 – 25% 26 – 50% 51 – 75% >75%

13 – 17 years 0% 1 – 25% 26 -50% 51 – 75% >75%

18 - 35 years 0% 1 – 25% 26 – 50% 51 – 75% >75%

>35 years 0% 1 – 25% 26 – 50% 51 – 75% >75%

12. Please circle the approximate percentage of your patient population in each category:

Cleft lip 0% 1 – 25% 26 – 50% 51 – 75% >75%

Cleft palate 0% 1 -25% 26 – 50% 51 – 75% >75%

Cleft lip and palate 0% 1 – 25% 26 – 50% 51 – 75% >75%

13. Please circle the approximate percentage of your patient population in each category:

Unoperated cleft palate 0% 1 – 25% 26 – 50% 51 – 75% >75%

Operated cleft palate 0% 1 – 25% 26 – 50% 51 – 75% >75%

Prosthetic management 0% 1 – 25% 26 – 50% 51 – 75% >75%

Pharyngeal flap surgery 0% 1 – 25% 26 – 50% 51 – 75% >75%

14. Please circle the approximate percentage of the types of services you provide monthly

Evaluation 0% 1 – 25% 26 – 50% 51 – 75% >75%

Treatment 0% 1 – 25% 26 – 50% 51 – 75% >75%

Support services 0% 1 – 25% 26 – 50% 51 – 75% >75%

Research 0% 1 – 25% 26 – 50% 51 – 75% >75%

Referral/source 0% 1 – 25% 26 – 50% 51 – 75% >75%

15. What has been your greatest challenge in the treatment of cleft patients?

Finance………….., logistics…………., socio cultural………, others(specify)………
